# Randomized, double-blind, placebo-controlled, crossover study of the efficacy and safety of lisdexamfetamine dimesylate in adults with attention-deficit/hyperactivity disorder: novel findings using a simulated adult workplace environment design

**DOI:** 10.1186/1744-9081-6-34

**Published:** 2010-06-24

**Authors:** Timothy Wigal, Matthew Brams, Maria Gasior, Joseph Gao, Liza Squires, John Giblin

**Affiliations:** 1 Department of Pediatrics, University of California, Irvine, Child Development Center, Irvine, CA, USA; 2 Private Practice, Bayou City Research, Houston, TX, USA; 3 Global Clinical Medicine, Shire Development Inc., Wayne, PA, USA; 4 Biostats, Shire Development Inc., Wayne, PA, USA; 5 Clinical Study Centers, LLC, Little Rock, AR, USA

## Abstract

**Background:**

Duration of efficacy and safety of lisdexamfetamine dimesylate (LDX) was assessed in adults (18-55 years) with attention-deficit/hyperactivity disorder (ADHD) using the simulated adult workplace environment.

**Methods:**

After open-label dose optimization (4-week) with LDX, 30-70 mg/d, subjects entered a 2-week randomized, double-blind, placebo-controlled crossover phase. Efficacy assessments included the Permanent Product Measure of Performance (PERMP) total score (attempted+correct) measured predose and from 2 to 14 hours postdose, averaged across postdose sessions (primary) and at each time point vs placebo (secondary), and ADHD Rating Scale IV (ADHD-RS-IV) with adult prompts at baseline and crossover visits. Safety assessments included treatment-emergent adverse events (TEAEs), vital signs, and electrocardiograms.

**Results:**

Of 127 randomized subjects, 105 were in the intention-to-treat population and 103 completed the study. While receiving LDX vs placebo, adults had greater improvement (*P *< .0001) in average PERMP total scores as measured by difference in least squares (LS) mean (95% CI): 23.4 (15.6, 31.2). Absolute (*P *≤ .0017 for each time point) and change from predose (*P *< .001 for each time point) PERMP total scores were greater at all postdose time points from 2 to 14 h for adults while receiving LDX vs placebo. LDX demonstrated efficacy vs placebo (*P *< .0001) by the difference in LS mean (95% CI) for ADHD-RS-IV total scores: -11.5 (-14.2, -8.9). TEAEs (≥ 10%) during dose optimization were decreased appetite, dry mouth, headache, and insomnia; no TEAEs ≥ 5% were reported during crossover phase for adults receiving LDX.

**Conclusions:**

LDX significantly improved PERMP scores vs placebo and maintained improvement throughout the day from the first (2 hours) to last (14 hours) postdose time point vs placebo in adults with ADHD.

**Trial Registration:**

ClinicalTrials.gov Identifier: NCT00697515

Safety and Efficacy Workplace Environment Study of Lisdexamfetamine Dimesylate (LDX) in Adults With Attention-Deficit Hyperactivity Disorder (ADHD) http://www.clinicaltrials.gov/ct2/show/NCT00697515?term=NCT00697515&rank=1

## Background

Attention-deficit/hyperactivity disorder (ADHD) in adults has an estimated prevalence of 4.4% in the United States and 3.4% worldwide [1,2]. Adults with ADHD experience significant impairment [1,3] in multiple domains of daily living, including the workplace, home, and various social settings [3,4].

For many years, pharmacotherapy has been recognized as having an important role in reducing the core symptoms of ADHD in adults [5]. Long-acting oral stimulants [6-8] have demonstrated efficacy in managing ADHD symptoms in adults [7,9-12]. However, in a survey study completed over a 12-month period in 2004, the prevalence of treatment for ADHD in adults was only 10.9% [1].

Lisdexamfetamine dimesylate (LDX) is a long-acting prodrug stimulant indicated for the treatment of ADHD in children 6 to 12 years of age and in adults in the United States. LDX is a therapeutically inactive molecule. Following oral ingestion, LDX is converted to l-lysine and active d-amphetamine. While a small amount of LDX is hydrolyzed to d-amphetamine in the gastrointestinal tract, the conversion of LDX into active d-amphetamine occurs primarily in the blood. The combination of l-lysine and d-amphetamine created a new chemical entity (a prodrug) with sustained delivery of d-amphetamine [13,14]. LDX demonstrated efficacy compared with placebo by the Permanent Product Measure of Performance (PERMP) and other assessments in the laboratory school setting at 12 and 13 hours postdose in children with ADHD [15,16]. In another pediatric study, LDX was effective throughout the day, as measured by parent ratings [17]. In these studies, LDX demonstrated a safety profile consistent with long-acting stimulant use [15-17].

LDX was also effective, with typically mild to moderate adverse events (AEs), in a large placebo-controlled trial in adults with ADHD [18]. Common AEs with LDX in this study included decreased appetite, dry mouth, and insomnia [18]. Efficacy was assessed through weekly evaluations of the ADHD Rating Scale IV (ADHD-RS-IV) with adult prompts and the Clinical Global Impressions (CGI) scale. Ratings of efficacy during the course of the day were not assessed in the initial study [18].

While the factors that determine treatment and choice of pharmacotherapy are complex, there may be a clinical need for long-acting stimulant medication with efficacy beyond 12-hours duration among adults with ADHD who require symptom control that extends throughout the day and into evening home and family time [19,20]. To assess and document the duration of efficacy of LDX throughout the day in adults with ADHD, the present study compared LDX with placebo in the simulated adult workplace environment (AWE) setting. Assessments, including AEs, vital signs, electrocardiogram (ECG), and physical examination, evaluated the safety profiles of the 2 treatment arms.

The simulated AWE is a structured, controlled environment based on the model of the laboratory school protocol (LSP) [21], designed to monitor and quantitatively assess response to medication in the performance of adults during activities simulating those that occur during a typical work day [21]. The use of the LSP, and specifically the PERMP assessment, has been applied widely to evaluate the effects of long-acting stimulants for children with ADHD [15,22-26]. Although not included in this study analysis, the LSP may include additional behavioral assessments such as a revised form of the Swanson, Kotkin, Agler, M-Flynn, and Pelham (SKAMP) rating scale and/or subject-reported behavioral assessment [4] to evaluate onset and duration of medication effects with validated, quantitative, and reproducible measures [21].

The simulated AWE is a useful tool for measuring attention and behavior because structured activities, designed to provoke behaviors associated with ADHD symptoms, are provided throughout the day and yield quantifiable outcomes. PERMP [21], a skill-matched test consisting of simple math problems to be attempted and completed at multiple time points throughout the simulated AWE session, is used to measure the ability to stay on task and attend to work. This instrument measures how effectively a subject initiates, self-monitors, and completes written seatwork [21]. It is not a test of the ability to learn math since the difficulty of problems is adjusted to the existing math skill level of each subject at baseline to ensure that each individual achieves ≥ 95% correct solutions. The PERMP is a validated, time sensitive, skill adjusted math test that measures attention in ADHD.

The goal of this study was to evaluate the efficacy of LDX compared with placebo in adults with ADHD in the simulated AWE setting, and to assess the duration of effect in a highly structured, controlled environment from 2 to 14 hours postdose.

## Methods

### Subjects

Adults (aged 18 to 55 years) with a primary diagnosis of ADHD were enrolled, based on criteria outlined in the *Diagnostic and Statistical Manual of Mental Disorders, Fourth Edition, Text Revision *(DSM-IV-TR™). ADHD diagnosis was further validated by a comprehensive psychiatric evaluation that included a semi-structured interview based on the Adult ADHD Clinical Diagnostic Scale, version 1.2 (ACDS v1.2) [27]. All subjects were also required to have scores on the ADHD-RS-IV with adult prompts ≥ 28 at baseline and a level of intellectual functioning equivalent to an intelligence quotient of ≥ 80 on the Kaufman Brief Intelligence Test [28]. Key exclusion criteria were the presence of a comorbid psychiatric diagnosis with significant symptoms, a history of, or perceived risk for future suicide attempt, a recent history of substance abuse, or other medical conditions that would contraindicate treatment with psychostimulants or confound efficacy and safety assessments. Exclusion criteria also included a history of seizures; hypertension, with a resting systolic blood pressure (SBP) > 139 mm Hg or diastolic blood pressure (DBP) > 89 mm Hg; or a history of symptomatic cardiovascular disease; a structural cardiac abnormality; or a positive family history of sudden cardiac death or ventricular arrhythmia. Other exclusion criteria included adverse reactions or lack of response to previous amphetamine therapy, concomitant medications affecting the central nervous system or blood pressure (with the exception of ADHD medications that were washed out), pregnancy or lactation, a body mass index < 18.5 and ≥ 40, or a clinically significant laboratory or ECG abnormality. Subjects whose current ADHD medication provided effective control of symptoms with acceptable tolerability were also excluded.

### Study setting

The simulated AWE is a controlled environment based on the LSP, but modified for the adult 14-hour day. Subjects arrived at 6 AM and departed at approximately 9:30 PM for both AWE sessions. The 2 AWE sessions, spaced 1 week apart in the double-blind phase, were organized into 3 sequential classes; each class consisting of a scheduled series of activities was designed to provoke all of the DSM-IV-TR™ symptoms of ADHD and to further provide objective measures of subject performance. In contrast to the child analog classroom design, the adult design is less reliant on behavioral observations and primarily focuses on objective measures (eg, PERMP math test). In the adult study, other mandatory activities and assignments were scheduled throughout the day, designed to provoke specific ADHD symptoms and were collected but not recorded as measurable assessments. Each classroom session included several 5-minute transition periods, a 10-minute PERMP test, and 10-minute academic group games. Key activities performed throughout the simulated AWE day included the presentation of a brief instructional video on a topic of general information followed by a factual quiz, time estimation tasks, practical checkbook balancing vignettes, and simple grammar error search tasks. Results from these activities were not analyzed as formal outcome assessments, but were included in the AWE day in order to actively involve subjects with effortful, repetitive, and uninteresting tasks provided to challenge subjects and thereby provoke the usual symptoms of ADHD [21].

### Study design

This randomized, double-blind, placebo-controlled, 2-way crossover study with an open-label dose-optimization phase was conducted in a simulated AWE. It was designed to assess duration of efficacy, tolerability, and safety of LDX (Vyvanse^®^, Shire US Inc.) (30, 50, and 70 mg/d) in adults with ADHD. This study was conducted at 5 centers in the United States.

This study was conducted in accordance with the Declaration of Helsinki and Good Clinical Practice according to the International Conference on Harmonisation guidelines. The study protocol was approved by each center's institutional review board. After complete explanation of the study to the subjects, written consent was obtained.

**Figure 1:**
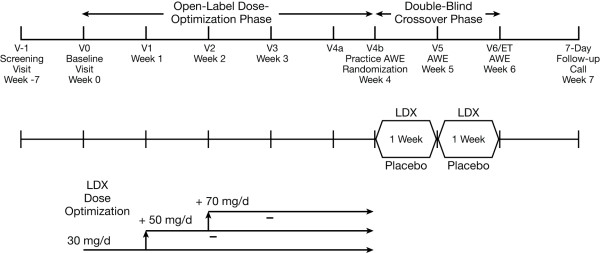
**Study design**. (AWE = adult workplace environment; LDX = lisdexamfetamine dimesylate).

The study comprised 4 phases: screening and washout (6 weeks); open-label dose optimization (4 weeks); double-blind crossover (2 weeks) which included 2 full-day evaluations in the simulated AWE; and a 7-day safety follow-up (Figure 1).

The primary objective was to evaluate the efficacy of LDX vs placebo by PERMP scores in adults with ADHD in the simulated AWE. A key secondary objective was to assess duration of effect over the day of LDX vs placebo in the simulated AWE with the PERMP administered at -0.5 hours predose and 2, 4, 8, 10, 12, and 14 hours postdose. Other objectives were to assess the efficacy of LDX for improvement in ADHD symptoms using the ADHD-RS-IV with adult prompts and to evaluate the improvement in ADHD symptom severity employing the CGI-Severity (CGI-S) scale at baseline and the CGI-Improvement (CGI-I) scale following LDX administration during the dose-optimization and double-blind phases.

#### Screening and washout

Except for stimulant medications and sedating antihistamines, which were discontinued 7 days prior to assessment of baseline measures, all prohibited medications were discontinued 30 days prior to screening. After washout, subjects returned to the clinic (baseline, visit 0) for reassessment of eligibility and to establish baseline safety and efficacy measures, including the ADHD-RS-IV with adult prompts, the CGI-S scale, vital signs, and ECG.

#### Open-label dose optimization

Following screening and washout, eligible subjects entered the open-label dose-optimization phase, during which they began receiving LDX and were evaluated for efficacy and tolerability at weekly visits. The dosage was initiated at 30 mg/d of LDX and upwardly titrated to the next available dose at weekly intervals until the optimal dose was reached. The optimal dose was defined as the dose that produced an overall minimum reduction in ADHD-RS-IV with adult prompts symptom score ≥ 30%, a CGI-I rating of 1 or 2, with tolerable side effects. Tolerability was determined by the investigator, based on a review of AEs and clinical judgment. Once reached, the optimal dose was maintained for the remainder of the dose-optimization phase and was used for the double-blind phase.

Overall response was assessed and categorized according to 3 possible conditions: intolerable response (presence of intolerable AEs); ineffective response (response to LDX of < 30% reduction from baseline in the ADHD-RS-IV score or a CGI-I rating > 2); and acceptable response (response with ≥ 30% reduction in the ADHD-RS-IV score and a CGI-I rating of 1 or 2, very much or much improved, but with tolerable AEs).

Subjects who experienced an intolerable response were permitted to be down-titrated only once by 20 mg/d to the next available lower dose. If dose reduction was tolerated and ADHD symptom control was acceptable, that dose was maintained for the remainder of the study. Subjects with an ineffective response were titrated to the next higher dose (eg, 50 or 70 mg/d), provided AEs were tolerable. For subjects with an acceptable response and tolerance of all prior doses, a further increase to achieve additional symptom reduction was permitted (to the maximum of 70 mg/d) at the clinician's discretion.

The last visit at which dosage adjustments could be made was visit 3 of the dose-optimization phase. For all randomized subjects, the dose dispensed at visit 3 was administered during the active week of the crossover phase. During week 4, visit 4a was to ensure quality-of-life assessments were completed, while visit 4b was for the practice AWE sessions.

#### Double-blind crossover phase

Subjects then entered a 2-week double-blind crossover phase and were randomized by a fixed-block randomization schedule to receive either their optimized dose of LDX for 7 days followed by placebo for 7 days or placebo for 7 days followed by their optimized dose of LDX for 7 days. On the last day of the first and second treatment sequence (visits 5 and 6), assessments of efficacy and safety of LDX or placebo were collected in the simulated AWE. Efficacy assessments collected during visits 5 and 6 were as follows: PERMP at -0.5 hour predose and 2, 4, 8, 10, 12, and 14 hours postdose; ADHD-RS-IV and CGI-I at specified times during the simulated AWE day, as well as safety assessments including weight measurements and a 12-lead ECG. Vital signs (ie, SBP, DBP, and pulse) were also collected at 1 hour predose and 4.5 and 14 hours postdose (± 45 minutes for each). A pregnancy test and a physical exam were performed during visit 6.

#### Follow-up

A follow-up by telephone was conducted 1 week after each subject's last dose of study drug to obtain information about any ongoing or new AEs or serious AEs and concomitant medications.

### Outcome measures

#### Efficacy measures

The primary efficacy endpoint, designed to evaluate the efficacy of LDX vs placebo, was the total PERMP scale scores averaged over all postdose time points assessed in AWE classroom sessions during visits 5 and 6. A secondary outcome measure, designed to evaluate the duration of effect of LDX vs placebo, was the total PERMP scores at each of the following time points: 2, 4, 8, 10, 12, and 14 hours postdose.

The PERMP, a 10-minute skill-adjusted math test, was used to evaluate effortful performance in the simulated AWE as a measure of treatment efficacy. The appropriate difficulty level for each subject for the PERMP was determined at screening based on results of a timed math pretest. The total PERMP score was the sum of the number of math problems attempted (PERMP-A) and the number of math problems answered correctly (PERMP-C) in a 10-minute session. The PERMP was completed throughout both AWE assessment days (visits 5 and 6).

The ADHD-RS-IV with adult prompts is a clinician-rated scale that assesses symptoms of ADHD based on DSM-IV-TR™ criteria [29]. The ADHD-RS-IV consists of 18 items that are grouped into 2 subscales: hyperactivity/impulsivity and inattention. Each item is scored on a scale of 0 (no symptoms) to 3 (severe symptoms), yielding a total score of 0 to 54 [29]. The ADHD-RS-IV was administered at baseline, visits 1 to 3 and visit 4b of the dose-optimization phase, and during the 2 AWE sessions, visits 5 and 6. The clinician-rated scale was administered by trained raters utilizing adult prompts developed at New York University and Massachusetts General Hospital [30,31].

The CGI provides a global evaluation of baseline severity and assesses improvement over time [32]. At baseline, the investigator used the CGI-S scale to rate severity of illness on a scale that ranged from 1 (normal, not at all ill) to 7 (among the most extremely ill subjects). At each visit thereafter (visits 1 to 3 and 4b of the dose-optimization phase and the 2 AWE sessions, visits 5 and 6 during the double-blind phase), the clinician used the CGI-I to rate improvement relative to baseline on a scale ranging from 1 (very much improved) to 7 (very much worse) [32].

### Safety

Safety assessments included monitoring AEs, concomitant medications, vital signs, 12-lead ECGs, and physical examination. At each study visit, AEs and concomitant medications were recorded. Resting SBP and DBP, pulse, temperature, weight, and respiratory rate were assessed at all study visits, except visit 4a. During AWE days (visit 5 and 6), SBP, DBP, and pulse were assessed at 3 specified time points. ECGs were conducted at screening, baseline, and visits 5 and 6. A physical examination was conducted at screening, baseline, and the end-of-study visit. The vital signs and ECG results were summarized according to the actual dose received. Treatment-emergent AEs (TEAEs), referring to events with onset after the first date of treatment, and no later than 3 days following termination of treatment, were recorded separately for the dose-optimization and the double-blind crossover phases of the study. TEAEs that continued uninterrupted from the dose-optimization to the crossover phase without a change in severity were counted only in the dose-optimization phase category. TEAEs with a change in severity across phases or that resolved and then restarted in the crossover phase were counted both in the dose-optimization and crossover arms. TEAEs for which a missing or incomplete start date made it impossible to determine in which phase of the study they started were counted as starting in the dose-optimization phase. TEAEs were reported as number and percentage of subjects according to system-organ class, preferred term, treatment group, and by last dose received at AE onset. AEs were collected at all visits by soliciting subject report with nonleading questions, and were coded using the *Medical Dictionary for Regulatory Activities *(MedDRA).

### Statistics

Based on estimates from earlier simulated AWE and pediatric laboratory school studies, the ratio between LDX/placebo differences and within-subject standard deviation was anticipated to be ≥ 0.49 at the 14-hour postdose time point, necessitating 90 subjects to complete the study to achieve 90% power for a 2-tailed test at the significance level of 0.05. With an anticipated dropout rate of 15% abstracted from previous studies, 106 subjects were targeted for enrollment.

The intention-to-treat population, defined as subjects who were randomized and had ≥ 1 primary efficacy measurement (average postdose PERMP total) collected, was used for primary efficacy analysis of PERMP scores. A linear mixed effects analysis of variance model, including treatment, period, and sequence as fixed effects and subjects as a random effect, was used for the primary efficacy analysis. All efficacy tests were conducted as 2-sided and at the significance level of 0.05. Two-sided confidence intervals were constructed with 95% coverage. No imputation of missing data was performed for the PERMP assessments. For other secondary efficacy measures, missing scores were imputed if the number of missing items was < 20% of the total number of items in the scale or subscale.

Due to the small and varied number of subjects enrolled per site and the within-subject design of statistical analyses in this study, analysis by site was not performed and site was not included as a factor in inferential analyses.

CGI-I ratings are reported in 2 dichotomized groups: improved, comprising very much and much improved (CGI-I ratings of 1 or 2), and not improved, comprising all other scores (CGI-I ratings of ≥ 3) excluding scores of 0 (not assessed). Prescott's test was used to compare dichotomized CGI-I outcomes during the crossover phase.

The safety population included all subjects who entered the dose-optimization phase and received ≥ 1 dose of LDX, and the randomized safety population included all subjects who were randomized and received ≥ 1 dose of blinded study drug during the double-blind crossover phase.

## Results

### Demographics and disposition

The study enrolled 142 subjects from 5 study centers (n = 36, 33, 28, 33, and 12) in the United States and was conducted from July to December 2008. All enrolled subjects were included in the safety population. The demographics appeared generally balanced between final dose levels in the dose-optimization phase (Table 1). The safety population had a mean age of 30.5 years and was predominantly white (89.4%), and male (62.0%), with a predominant combined ADHD subtype (69.0%). Of enrolled subjects, 127 were randomized and administered a dose of study medication in the crossover phase, 103 (72.5%) completed the study, and 39 (27.5%) discontinued prematurely (Table 2). No subject discontinued due to lack of efficacy, whereas 6 subjects attributed withdrawal to TEAEs. Of the subjects who withdrew during the dose-optimization phase due to TEAEs, 3 withdrew due to elevated blood pressure, and 1 due to cardiac arrhythmia (frequent premature ventricular complexes). Two subjects withdrew due to TEAEs during the crossover phase: 1 for gastroenteritis and 1 for viral infection. Additionally, 17 subjects withdrew prematurely from the study during the crossover phase because their study participation coincided with the occurrence of a natural disaster (ie, hurricane) in the vicinity of the participating study site, leading to site closure (listed as Other in Table 2).

**Table 1:** Demographic and baseline characteristics (safety population) by last dose in the dose-optimization phase

Characteristic, mean (SD)	LDX 30 mg/d (n = 28)	LDX 50 mg/d (n = 70)	LDX 70 mg/d (n = 44)	LDX All Doses (N = 142)
Age (years)	30.5 (9.54)	29.7 (10.71)	31.8 (11.46)	30.5 (10.70)

Weight (lb)	174.7 (43.64)	176.7 (35.22)	182.4 (36.17)	178.1 (37.14)

Height (in)	65.8 (4.30)	68.3 (3.73)	68.3 (3.77)	67.8 (3.97)

Body mass index (lb/in^2^)	28.2 (5.45)	26.6 (4.79)	27.4 (5.10)	27.2 (5.02)

Gender, n(%)				

Male/female	16 (57.1)/12 (42.9)	45 (64.3)/25 (35.7)	27 (61.4)/17 (38.6)	88 (62.0)/54 (38.0)

Race, n (%)				

White	21 (75.0)	65 (92.9)	41 (93.2)	127 (89.4)

Black/African American	4 (14.3)	2 (2.9)	0	6 (4.2)

Native Hawaiian/Pacific Islander	2 (7.1)	0	0	2 (1.4)

Asian	1 (3.6)	2 (2.9)	2 (4.5)	5 (3.5)

American Indian/Alaskan Native	0	0	1 (2.3)	1 (0.7)

Other	0	1 (1.4)	0	1 (0.7)

Ethnicity, n (%)				

Hispanic or Latino/ Not Hispanic or Latino	2 (7.1)/26 (92.9)	7 (10.0)/63 (90.0)	4 (9.1)/40 (90.9)	13 (9.2)/129 (90.8)

ADHD subtype, n (%)				

Inattentive	8 (28.6)	20 (28.6)	11 (25.0)	39 (27.5)

Hyperactive/impulsive	1 (3.6)	3 (4.3)	1 (2.3)	5 (3.5)

Combined	19 (67.9)	47 (67.1)	32 (72.7)	98 (69.0)

ADHD-RS-IV with adult prompts: scores at baseline, mean (SD)				

Total	37.8 (6.06)	35.8 (4.85)	38.4 (6.12)	37.0 (5.61)

Inattentive	20.9 (3.15)	19.7 (3.45)	20.8 (3.66)	20.3 (3.49)

Hyperactivity/impulsivity	16.9 (5.52)	16.1 (4.35)	17.6 (4.86)	16.7 (4.77)

ADHD = attention-deficit/hyperactivity disorder; ADHD-RS-IV = ADHD Rating Scale IV; LDX = lisdexamfetamine dimesylate.

**Table 2:** Subject disposition by treatment sequence

		Randomization Sequence		
**n (%)**	**Discontinued Prior to Randomization** **(n = 15)**	**LDX/Placebo** **(n = 63)**	**Placebo/LDX** **(n = 64)**	**All Subjects** **(N = 142)**

Safety population	15 (100.0)	63 (100.0)	64 (100.0)	142 (100.0)

Randomized safety population	-	63 (100.0)	64 (100.0)	127 (89.4)

Intention-to-treat population	-	53 (84.1)	52 (81.3)	105 (73.9)

Per protocol population	-	49 (77.8)	49 (76.6)	98 (69.0)

Completed study	-	52 (82.5)	51 (79.7)	103 (72.5)

Discontinuations	15 (100.0)	11 (17.5)	13 (20.3)	39 (27.5)

Reasons for discontinuations				

Total	15 (100.0)	11 (17.5)	13 (20.3)	39 (27.5)

AE	4 (26.7)	0	2 (3.1)	6 (4.2)

Lack of efficacy	0	0	0	0

Refused further participation	5 (33.3)	3 (4.8)	2 (3.1)	10 (7.0)

Protocol nonadherence/subject noncompliant	0	0	0	0

Lost to follow-up	2 (13.3)	0	0	2 (1.4)

Other	4 (26.7)	8 (12.7)^a^	9 (14.1)^a^	21 (14.8)

^a^Seventeen subjects withdrew from the study during the double-blind crossover phase due to a natural disaster-related study site closure (ie, hurricane).

AE = adverse event; LDX = lisdexamfetamine dimesylate.

### Efficacy

**Figure 2:**
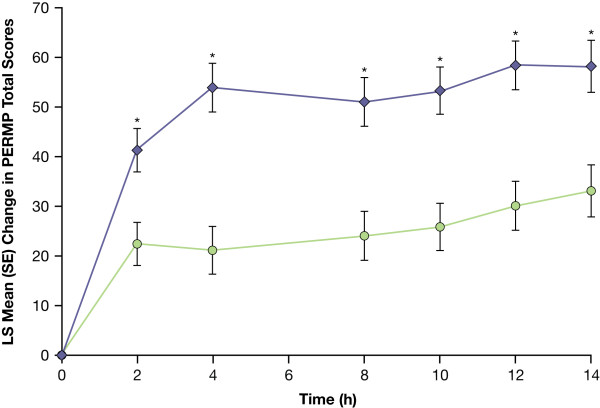
**LS Mean (SE) change from predose in PERMP total score from 2 to 14 hours postdose (n = 104/104)**. (LDX = lisdexamfetamine dimesylate; LS = least squares; PERMP = Permanent Product Measure of Performance). LDX-purple diamonds; Placebo-green circles. * *P *< .001 LDX vs placebo.

**Figure 3:**
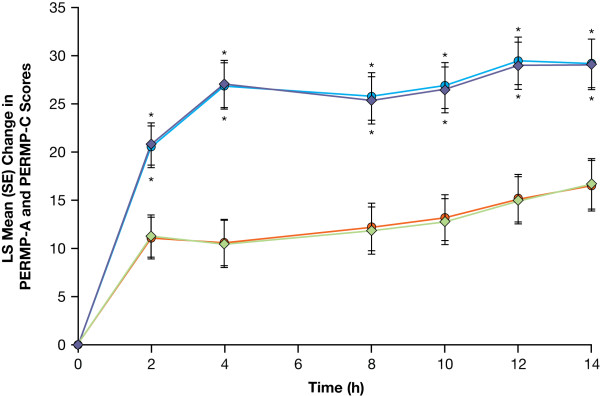
**LS mean (SE) change from predose in PERMP-A and PERMP-C scores from 2 to 14 hours postdose (n = 104/104)**. (LDX = lisdexamfetamine dimesylate; LS = least squares; PERMP-A/-C = Permanent Product Measure of Performance-Attempted/-Correct). PERMP-A: LDX-purple diamonds; Placebo-green diamonds; PERMP-C: LDX-blue circles; Placebo-orange circles.* *P *< .001 LDX vs placebo.

LDX demonstrated efficacy vs placebo on the primary endpoint, the average total PERMP score from all postdose assessments during the AWE sessions. The mean average postdose PERMP total score was significantly greater (F = 35.47; df [1, 101]; *P *< .0001) for adults while receiving LDX vs placebo (Table 3). PERMP was assessed in 105 subjects, with 1 subject withdrawing from each of the 2 treatment sequences after the first crossover assessment period (visit 5). Analysis of absolute values of total PERMP scores at postdose time points demonstrated significant efficacy for adults while receiving LDX vs placebo at each time point (*P *≤ .0017). LS mean (SE) change from predose of the PERMP total score (n = 104) for adults while receiving LDX demonstrated significant (*P *< .001) efficacy vs placebo at all time points evaluated from 2 hours to 14 hours postdose (Figure 2). Improvement in average postdose scores was also significantly greater (*P *< .0001) for adults receiving LDX vs placebo for PERMP-A and PERMP-C scores as measured by the difference in LS mean (Table 3). Analysis of absolute values of PERMP-A and PERMP-C scores at postdose time points demonstrated significant efficacy for adults while receiving LDX vs placebo at each time point (*P *≤ .0031). LDX also demonstrated efficacy vs placebo as measured by change from predose at each postdose time point from 2 hours to 14 hours with higher LS mean (SE) PERMP-A and PERMP-C scores for LDX vs placebo (Figure 3).

**Table 3:** Predose and average postdose PERMP scores: PERMP total, PERMP-A, and PERMP-C (n = 104)

	Predose PERMP Mean (SD)	Average Postdose PERMP Mean (SD)	Difference in Postdose LS Mean (95% CI) (LDX/Placebo)	*P *Value
PERMP total				

While receiving LDX	260.1 (86.23)	312.7 (94.42)	23.4 (15.6, 31.2)	< .0001
		
While receiving placebo	261.4 (74.96)	287.6 (81.45)		

PERMP-A				

While receiving LDX	132.2 (43.28)	158.4 (47.53)	12.0 (8.1, 15.8)	< .0001
		
While receiving placebo	132.6 (37.62)	145.7 (41.06)		

PERMP-C				

While receiving LDX	127.9 (43.02)	154.3 (46.96)	11.5 (7.6, 15.4)	< .0001
		
While receiving placebo	128.8 (37.39)	141.9 (40.44)		

CI = confidence interval; LDX = lisdexamfetamine dimesylate; LS = least squares; PERMP = Permanent Product Measure of Performance; PERMP-A = PERMP-Attempted; PERMP-C = PERMP-Correct.

During the open-label dose-optimization phase with all subjects receiving LDX, ADHD-RS-IV total scores decreased. At baseline, mean (SD) ADHD-RS-IV total scores were 37.0 (5.61). At visits 1, 2, 3, 4b and at dose-optimization endpoint, mean (SD) change from baseline scores were -12.3 (8.32), -16.8 (7.83), -20.6 (7.07), -21.6 (7.40), and -21.4 (7.31), respectively (*P *< .0001). Decreases from baseline to dose-optimization endpoint were also shown with ADHD-RS-IV inattention and hyperactivity/impulsivity subscores (*P *< .0001 for each). Mean (SD) ADHD-RS-IV inattention subscores were 20.3 (3.49) at baseline, and mean (SD) change from baseline at dose-optimization endpoint was -11.6 (4.33). Mean (SD) ADHD-RS-IV hyperactivity/impulsivity subscores were 16.7 (4.77) at baseline, and mean (SD) change from baseline at dose-optimization endpoint was -9.8 (4.38).

**Figure 4:**
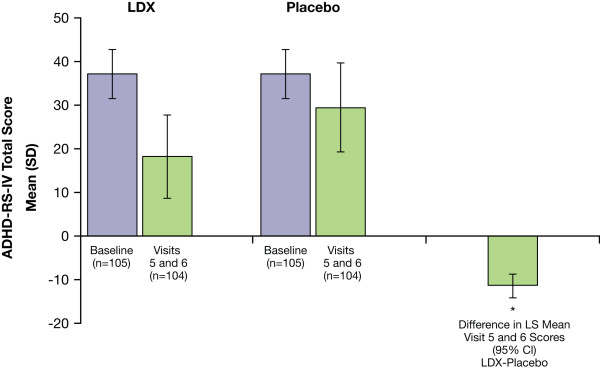
**ADHD-RS-IV total scores at baseline and visit 5/6 and difference in LS mean (95% CI) between LDX and placebo during the double-blind crossover phase**. (ADHD-RS-IV = Attention-Deficit/Hyperactivity Disorder Rating Scale IV; CI = confidence interval; LDX = lisdexamfetamine dimesylate; LS = least squares). * *P *< .0001 LDX vs placebo.

The mean (SD) percent change in ADHD-RS-IV total scores from baseline at visits 5 and 6 (double-blind, crossover period) for adults while receiving LDX (all doses) was -51.5% (24.24) and while receiving placebo was -21.3% (24.41). The mean ADHD-RS-IV total score was significantly lower, indicating better symptom control, for adults while receiving LDX vs placebo (*P *< .0001) as measured by the differences (LDX vs placebo) in LS mean ADHD-RS-IV total scores during visits 5 and 6 (Figure 4). Significant improvements for adults while receiving LDX vs placebo were also seen for inattention and hyperactivity/impulsivity subscales as measured by the difference in LS mean scores (95% CI) during visits 5 and 6: -6.3 (-7.7, -4.9; *P *< .0001) for inattention scores and -5.2 (-6.6, -3.7; *P *< .0001) for hyperactivity-impulsivity scores.

At baseline, all subjects (n = 142) were rated moderately (64.8%), markedly (32.4%), or severely (2.8%) ill by CGI-S with a mean (SD) score of 4.4 (0.5). During the double-blind crossover phase, CGI-I ratings suggested that 88 (76.5%) of 115 subjects improved while taking LDX (all doses) and 27 (23.1%) of 117 subjects improved while taking placebo. For subjects with valid CGI-I ratings at both visits 5 and 6, of those randomized to the LDX/placebo sequence in the crossover phase, 27 of 52 subjects demonstrated improvement (much or very much improved on the CGI-I) only while receiving LDX; 9 improved only while receiving placebo. For subjects randomized to the placebo/LDX sequence, 43 of 51 subjects demonstrated improvement only while receiving LDX and 4 improved while only on placebo. LDX was associated with significantly (*P *< .0001) lower CGI-I ratings vs placebo in the crossover phase (Prescott's test).

### Safety

No deaths or serious AEs were reported in this study. Most reported TEAEs were mild and moderate in severity. During the dose-optimization phase, the most common TEAEs of decreased appetite, dry mouth, headache, insomnia, upper respiratory tract infection, irritability, nausea, anxiety, and feeling jittery were reported by ≥ 5% of subjects (Table 4).

**Table 4:** TEAEs during the dose-optimization phase for all TEAEs with incidence ≥ 5% in either the dose-optimization and/or the crossover phases

AE Preferred Term, % (n)	Dose-Optimization Phase (Safety Population)
	
	LDX-All Doses (n = 142)^a^
Any TEAE	79.6 (113)

Anxiety	5.6 (8)

Decreased appetite	36.6 (52)

Dry mouth	30.3 (43)

Fatigue	4.9 (7)

Feeling jittery	5.6 (8)

Headache	19.7 (28)

Insomnia	18.3 (26)

Irritability	8.5 (12)

Nausea	7.7 (11)

Upper respiratory tract infection	9.9 (14)

TEAEs were assigned to either the open-label dose-optimization phase or the double-blind crossover phase of the study and were summarized separately. TEAEs that continued uninterrupted from the dose-optimization to the crossover phase without a change in severity were counted only in the dose-optimization phase category. TEAEs with a change in severity across phases or that resolved and then restarted in the crossover phase were counted both in the dose-optimization and crossover arms. TEAEs for which a missing or incomplete start date made it impossible to determine in which phase of the study they started were counted as starting in the dose-optimization phase.

^a^Percentages are based on the number of subjects who received each dose at any point during the dose-optimization phase.

AE = adverse event; LDX = lisdexamfetamine dimesylate, TEAE = treatment-emergent adverse event.

During the crossover phase, the overall incidence of TEAEs was greater while subjects received placebo vs LDX (Table 5). There were no TEAEs that were reported by ≥ 5% of subjects while receiving LDX. The TEAEs of fatigue and upper respiratory tract infection were reported by ≥ 5% of subjects while receiving placebo, which contributed to the increased incidence of TEAEs in subjects receiving placebo vs LDX (12.0% vs 0.9%, respectively, for fatigue and 7.7% vs 1.7%, respectively, for upper respiratory tract infection).

**Table 5:** TEAEs during the crossover phase for all TEAEs with incidence ≥ 5% in either the dose-optimization and/or the crossover phases

AE Preferred Term, % (n)	Crossover Phase (Randomized Population)	
	
	LDX-All Doses (n = 115)^a^	Placebo (n = 117)^a^
Any TEAE	27.8 (32)	35.9 (42)

Anxiety	1.7 (2)	0

Decreased appetite	3.5 (4)	1.7 (2)

Dry mouth	3.5 (4)	0.9 (1)

Fatigue	0.9 (1)	12.0 (14)

Feeling jittery	0	0

Headache	1.7 (2)	2.6 (3)

Insomnia	2.6 (3)	1.7 (2)

Irritability	0	0.9 (1)

Nausea	1.7 (2)	0

Upper respiratory tract infection	1.7 (2)	7.7 (9)

TEAEs were assigned to either the open-label dose-optimization phase or the double-blind crossover phase of the study and were summarized separately. TEAEs that continued uninterrupted from the dose-optimization to the crossover phase without a change in severity were counted only in the dose-optimization phase category. TEAEs with a change in severity across phases or that resolved and then restarted in the crossover phase were counted both in the dose-optimization and crossover arms. TEAEs for which a missing or incomplete start date made it impossible to determine in which phase of the study they started were counted as starting in the dose-optimization phase.

^a^Percentages are based on the number of subjects who received each dose at any point during the crossover phase.

AE = adverse event; LDX = lisdexamfetamine dimesylate, TEAE = treatment-emergent adverse event.

At baseline, the mean (SD) for SBP, DBP, and pulse were 119.6 (10.28) mm Hg, 73.8 (7.87) mm Hg, and 72.4 (11.23) bpm, respectively. At the endpoint of the dose-optimization phase, the mean (SD) for SBP, DBP, and pulse were 119.3 (10.40) mm Hg, 73.6 (7.65) mm Hg, and 75.6 (9.80) bpm, respectively. During the double-blind crossover phase, the mean at predose and postdose time points on visits 5 and 6 of the AWE days ranged from 118.0 to 120.5 mm Hg for SBP, 71.5 to 73.7 mm Hg for DBP, and 71.4 to 74.8 bpm for pulse in subjects while receiving placebo; and from 117.2 to 123.4 mm Hg for SBP, 73.3 to 75.5 mm Hg for DBP, and 77.0 to 81.0 bpm for pulse in subjects while receiving LDX (all doses). Consistent with prior clinical studies of LDX, ECG interval data showed no clinically meaningful trends. At baseline, the mean (SD) QTcF interval was 384.8 (19.68) msec and during the double-blind crossover phase (visits 5 and 6) was 388.8 (20.65) msec for subjects while receiving LDX, and the mean (SD) QTcF interval at visits 5 and 6 was 389.0 (21.52) for subjects while receiving placebo.

The mean (SD) change in weight at dose-optimization endpoint vs baseline was -4.0 (4.27) lb. During the crossover phase, the mean (SD) change in weight vs baseline for subjects administered placebo was -2.7 (3.98) and for subjects administered LDX was -4.4 (4.72) lb. The incidence of subjects who experienced a decrease in weight that was categorized as a TEAE (based on subject's self-report and clinician's judgment) was 3.5% (5 of 142 subjects) during the dose-optimization phase with none in the crossover phase.

## Discussion

This is the first study of a medication approved for the treatment of ADHD to examine efficacy and safety in adults with ADHD in a structured setting (ie, simulated AWE) where objective measures of efficacy could be assessed throughout the day and the first to demonstrate efficacy (vs placebo) of an approved oral stimulant medication at 14 hours postdose. While similar studies in adults are limited, efficacy of long-acting stimulants in the laboratory school setting has been demonstrated for children with ADHD across the day and at 12 [15,22,25,33,34] and 13 hours postdose [16]. The findings of the current study align closely with the results seen in children in a laboratory school setting [16]. In both studies, LDX demonstrated significant separation from placebo through the last postdose time points assessed on an objective measure of task productivity and accuracy throughout the day.

In this study, LDX demonstrated efficacy compared with placebo as measured by the average postdose PERMP math test total scores in this controlled trial in the simulated AWE setting. Moreover, LDX exhibited efficacy at all time points measured during the AWE sessions: from 2 hours to 14 hours postdose. Since ADHD symptoms may extend late into the day [20], the availability of treatments that provide efficacy throughout the day, is important.

LDX demonstrated efficacy compared with placebo in this study in decreasing symptoms of ADHD as measured by the ADHD-RS-IV with adult prompts. LDX also demonstrated efficacy based on improvements in global assessment of symptom severity as assessed by clinicians on the CGI-I scale. These findings support and extend previous findings that LDX reduced the symptoms and severity of ADHD compared with placebo in adults in a 4-week controlled trial [18] with measures assessed at weekly intervals (eg, CGI-I ratings and ADHD-RS-IV with adult prompts scores). In that randomized, forced-dose escalation, double-blind, placebo-controlled study for adult subjects with ADHD, LDX significantly reduced ADHD symptoms at each dose and at each weekly assessment beginning at week 1 and through study endpoint compared with placebo.

In the current study, LDX demonstrated a safety profile consistent with long-acting stimulant use. The common AEs in the current study, including decreased appetite, dry mouth, headache, and insomnia, are consistently seen in studies of long-acting stimulant medications administered to adults [12,18,35,36]. As demonstrated in these other studies, most AEs were mild to moderate in severity. The effects seen in the current study on weight and cardiovascular parameters were consistent with those previously reported for stimulants, including LDX, in adults [7-9,18,37]. As previously seen for LDX in adult patients with ADHD [18], LDX administration in the current study was associated with modest effects on cardiovascular parameters of blood pressure and pulse. Four subjects withdrew during dose optimization due to cardiovascular-related TEAEs, supporting the importance of monitoring cardiovascular parameters during treatment with stimulants. As with all stimulants, careful attention to cardiovascular history, symptoms, and clinical findings in adults with ADHD prior to, and during treatment with, stimulants is advisable.

Strengths of the study included experimental design features, such as the multicenter, double-blind, placebo-controlled, crossover design, and use of the simulated AWE setting and the validated PERMP to provide assessments of medication efficacy and safety compared with placebo throughout the day. While studies assessing the effects of treatment on symptom reduction over an extended time course (eg, weeks to months) is very useful in determining global efficacy and safety of medications for ADHD, it is also important to understand the effects of medications for ADHD in settings over the course of the day.

## Limitations

There are limitations on the interpretation of the results of this study. The duration of the study was relatively short. As an assessment of attention to task, ability to stay on task, and to monitor during repetitive task completion throughout the day, it should be kept in mind that, by its design, the PERMP math test setting may result in increased testing-related arousal. However, the simulated AWE, which includes multiple practice sessions and repeated testing sessions is designed to dampen such arousal. Additionally, the simulated AWE is intended to be analogous to real-world employment settings only in the sense of requiring adults to engage in activities that require attention, mental effort, and a quantifiable outcome (ie, written work). In this way the AWE is a setting to elicit ADHD symptoms that might manifest in a workplace where adults with ADHD are occupied with repetitive, effortful tasks. The exclusion of subjects with active cardiovascular conditions, other unstable medical conditions, or comorbid psychiatric disorders may limit the applicability of results to the clinically encountered population. Additionally, the expected dropout rate of 15% was exceeded because of an unexpected natural disaster (ie, hurricane) that resulted in the closure of 1 study site.

## Conclusions

LDX demonstrated consistent efficacy compared with placebo in a structured simulated AWE from 2 hours to 14 hours postdose as assessed by PERMP, a measure aimed at assessing attention, ability to stay on task, and to monitor tasks throughout the day. LDX was also efficacious in providing overall improvement in the majority of patients and demonstrated a safety profile consistent with long-acting stimulant use.

## Competing interests

TW financial disclosures are for the past 10 years and include research support, consulting honoraria, and/or speaker's bureau from the following pharmaceutical companies: Celltech, Cephalon, Eli Lilly, Janssen, McNeil, Novartis, Otsuka, and Shire. He has no stock or equity interests. Since July 2008, the list would be limited to Eli Lilly, McNeil, Otsuka, and Shire for either research support or consulting honoraria. In addition, he has received funding from NIMH, NICHD, and NIDA.

MB has been a speaker for Cephalon, Eli Lilly, McNeil, Novartis, Pfizer, Shire, and Wyeth.

MG is a full-time employee of Shire Development Inc.

JG is a full-time employee of Shire Development Inc.

LS is a full-time employee of Shire and has held stock and/or options in the following companies: Johnson & Johnson, Pfizer, and Shire.

JGn is/has receives/d research/grant support, is/has been on the speaker's bureau of or is/has been a consultant for the following pharmaceutical companies: Addrenex, Cephalon, GlaxoSmithKline, Johnson & Johnson, Novartis, Ortho-McNeil, Pfizer, Sanofi-Aventis, Sepracor, and Shire.

## Authors' contributions

TW was the principal investigator on this study, made substantial contributions to the conception and design of the study, enrolled patients, participated in data acquisition, analysis, interpretation, and presentation. He was deeply involved in drafting the manuscript and revising the intellectual content. He has given final approval of this version.

MB was an investigator on the study, enrolled patients, and participated in data acquisition, analysis, interpretation, and presentation. He was deeply involved in drafting the manuscript and revising the intellectual content. He has given final approval of this version.

MG was the Associate Director, Global Clinical Medicine for this study and made substantial contributions to the analysis and interpretation of the data. She was deeply involved in drafting the manuscript and revising the intellectual content. She has given final approval of this version.

JG was the statistician for this study and made substantial contributions to the analysis and interpretation of the data. He was deeply involved in drafting the manuscript and revising the intellectual content. He has given final approval of this version.

LS was the Senior Director, Global Clinical Medicine for this study and made substantial contributions to the analysis and interpretation of the data. She was deeply involved in drafting the manuscript and revising the intellectual content. She has given final approval of this version.

JGn was an investigator on the study, enrolled patients, and participated in data acquisition, analysis, interpretation, and presentation. He was deeply involved in drafting the manuscript and revising the intellectual content. He has given final approval of this version.
